# NLP2-NR Module Associated NO Is Involved in Regulating Seed Germination in Rice under Salt Stress

**DOI:** 10.3390/plants11060795

**Published:** 2022-03-17

**Authors:** Yake Yi, Yaqiong Peng, Tao Song, Siqiong Lu, Zhenning Teng, Qin Zheng, Fankai Zhao, Shuan Meng, Bohang Liu, Yan Peng, Guanghui Chen, Jianhua Zhang, Nenghui Ye

**Affiliations:** 1 College of Agriculture, Hunan Agricultural University, Changsha 410128, China; yykchn7@gmail.com (Y.Y.); 1123996324@stu.hunau.edu.cn (Y.P.); lusiqiong@stu.hunau.edu.cn (S.L.); znteng@cuhkri.org.cn (Z.T.); zhengqin@stu.hunau.edu.cn (Q.Z.); 17789369834@stu.hunau.edu.cn (F.Z.); mengshuanz@outlook.com (S.M.); liubohan@hunau.edu.cn (B.L.); pengyan3759@hunau.edu.cn (Y.P.); 2 Co-Innovation Center for Sustainable Forestry in Southern China, College of Biology and the Environment, Nanjing Forestry University, Nanjing 210037, China; songtao1987@njfu.edu.cn; 3 Department of Biology, Hong Kong Baptist University, Kowloon, Hong Kong 999077, China; 4 School of Life Sciences, State Key Laboratory of Agrobiotechnology, The Chinese University of Hong Kong, Shatin, Hong Kong 999077, China; 5 Hunan Provincial Key Laboratory of Rice Stress Biology, Hunan Agricultural University, Changsha 410128, China

**Keywords:** NIN-like protein 2, nitrate reductase, nitric oxide, salt stress, seed germination, abscisic acid, rice

## Abstract

Salt stress has the most severe impact on plant growth and development, including seed germination. However, little is known about the mechanism of NR (nitrate reductase)-associated nitric oxide (NO) regulates salt tolerance during seed germination in rice. Herein, we shown that inhibition of seed germination by salt stress was significantly impaired by sodium nitroferricyanide (SNP), a NO donor. Then a triple mutant, *nr1/nr2/nr3*, was generated. Results shown that germination of triple mutants were delayed and were much more sensitive to salt stress than WT plant, which can be rescued by application of SNP. qPCR analysis revealed that expressions of abscisic acid (ABA) catabolism gene, *OsABA8ox1*, was suppressed in triple mutants under salt stress, resulting in an elevated ABA content. Similar to SNP, application of nitrate also rescued seed germination under salt stress, which, however, was blocked in the triple mutants. Further study revealed that a nitrate responsive transcript factor, *OsNLP2*, was induced by salt stress, which thus up-regulates the expression of *OsNRs* and NR activity, resulting in promoted salt tolerance during seed germination. In addition, nitrate-mediated salt tolerance was impaired in mutant of *aba8ox1*, a target gene for NLP2. Transient trans-activation assays further revealed NLP2 can significantly activate the expression of *OsABA8ox1* and *OsNR1*, suggesting that NLP2 activates expression of ABA catabolism gene directly or indirectly via NR-associated NO. Taken together, our results demonstrate that NLP2-NR associated NO was involved in salt response by increasing ABA catabolism during seed germination and highlight the importance of NO for stress tolerance of plants.

## 1. Introduction

Soil salinity is one of the most devastating abiotic stresses. More than 20% of the cultivated lands are threatened by high salinity, which is aggravated by the growing population, poor agricultural practices, as well as salt intrusion in coastal zones, making salt stress an increasingly serious problem worldwide [1,2]. Salt stress can severely affect plant growth and development during the whole life-cycle, including seed germination, seedling establishment and yield formation [3]. To survive from this harsh environmental stress, plants have evolved different response mechanisms to deal with salt stress, such as salt tolerance, avoidance and escape, and recovery mechanism [4,5]. The SOS pathway plays a key role in regulating cellular ion homeostasis during salt stress and represents the well conserved salt tolerant mechanism in higher plants [3,6,7]. Plant hormones are another crucial regulator for plant growth in response to salt stress, among which ABA functions as a central integrator for developmental process and abiotic tolerance, including salt tolerance [8,9]. ABA plays a major and irreplaceable role during salt stress response in plants by regulating ion homeostasis, biosynthesis of osmolytes, ROS (reactive oxygen species) scavenging and salt-responsive gene expression. Application of exogenous ABA will impair the harmful effect of salt stress [10]. However, the content of ABA which is well known as a key repressor of seed germination [11] is increased by salt stress during seed germination [12]. Germination of an ABA biosynthesis defective mutants, such as *aba2*, was faster than that of WT under salt stress [12,13], suggesting that elevated ABA functions negatively in salt tolerance during seed germination.

Nitric oxide (NO) is a gaseous, free-radical and redox-signaling molecule which has multiple functions and acts as molecular messenger during plant growth and development. NO also plays important roles in response to different stresses, including drought stress, temperature stress, salt stress and heavy metal stress [14,15,16,17]. In plants, there are several origins for NO production, among which the nitrate reductase (NR) pathway is the best-characterized and most important pathway for NO production [18]. Double mutation of two NR encoding genes, *nia1/nia2*, resulted in reduced content of both NO and nitrite in *Arabidopsis* [19]. Numerous studies have proved that NO is critical in promoting salt tolerance of plant at different developmental stage [20,21]. In salt-affected plants, NO was found to significantly improve seed vigor and germination [22], while priming or pretreatment with SNP, a donor of NO, will significantly promote seed germination and early seedling growth under salt stress [23,24]. It has been well proved that NO play versatile roles in promoting salt tolerance, including maintaining ion homeostasis by enhancing K^+^ uptake [25], promoting antioxidant defenses and osmolytes biosynthesis and/or accumulation against to salinity-induced oxidative stress and osmotic stress, respectively [26,27], interacting with other signaling pathways in response to salt stress [28,29]. During seed germination, the accumulation of NO at early stage of germination resulted in degradation of ABA, which was blocked in *cyp707a2* mutant, a key gene for ABA catabolism [30]. Seed germination was inhibited in NR defective mutant, *nia1/nia2* [19], suggesting the importance of NR-associated NO during seed germination. However, it is still unclear whether the NR-associated NO also plays a role in response to salt stress during seed germination in rice.

Seed germination is a key developmental stage during the life cycle and is tightly controlled in plants [31,32]. Antagonistic interaction between ABA and GA (gibberellin) plays a crucial role in regulating seed germination [33]. During the early stage of seed germination, ABA catabolism was activated, which thus release the inhibiting effect on seed germination in rice. It has been demonstrated that nitrate was also involved in breaking seed dormancy and promoting seed germination independently of its reduction into NO by NR in *Arabidopsis* [34]. Recently, NIN-like protein (NLP8), a nitrate-activated transcription factor, was identified to activate the expression of *CYP707A2*, an ABA catabolism gene in *Arabidopsis*, and thus was essential for nitrate-promoted seed germination [35], suggesting that nitrate itself might act as a molecular signal to promote seed germination independent on NR-associated NO production. On the contrary, NLPs from rice were reported to activate the expression of *OsNR* and *OsNiR* genes and thus regulate the nitrogen use efficiency. OsNLP4 bind to the promoter of *OsNiR* gene and transactivate [36] while OsNLP1 and OsNLP3 bind to the promoter of *OsNR* genes and increase their abundance [37,38]. NR activity was elevated in over expressing plants of *OsNLP1* and *OsNLP3* but reduced in *nlp1* and *nlp3* mutants.

Moreover, both nitrate-promoted seed germination and stimulating effect on seedling growth by nitrate were blocked by application of NO-scavenger, 2-(4-carboxyphenyl)-4,4,5,5-tetramethylimidazoline-1-oxyl-3-oxide (c-PTIO) [39,40]. In addition, NR-dependent NO production was involved in promoting salt tolerance during seed germination in soybean [41] and in cucumber root [42] implying that nitrate promote plant development and improve stress tolerance is dependent on the NR-associated NO production. These results suggest that the regulating mechanism of seed germination under salt stress by NR-associated NO and nitrate in rice is still unclear.

In this study, we generated a triple mutant of three *OsNR* genes, which displayed a delayed germination phenotype and was much more sensitive to salt stress than seeds of WT plant. qPCR analysis revealed that expressions of ABA catabolism genes, *OsABA8ox1* and *OsABA8ox2*, were suppressed in triple mutants, especially under salt stress, resulting in an elevated ABA content. Similar to SNP, application of nitrate also promoted seed germination under salt stress, which, however, was blocked in the triple mutants. Further study revealed that a nitrate responsive transcript factor, *OsNLP2*, was induced by salt stress, which thus up-regulates the expression of *OsNRs* and NR activity, resulting in promoted salt tolerance during seed germination. In addition, nitrate-mediated salt tolerance was impaired in mutants of *aba8ox1* and *aba8ox2*, two target gene for NLP2, suggesting NLP2 might activate expression of ABA catabolism gene directly or indirectly via NR-associated NO. Altogether, our results demonstrate that NLP2-NR associated NO was involved in salt response by increasing ABA catabolism during seed germination and highlight the importance of NO for stress tolerance of plants.

## 2. Results

### 2.1. NR-Associated NO Is Involved in Enhancing Seed Germination under Salt Stress

**Figure 1:**
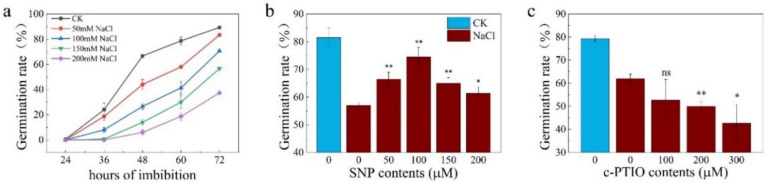
Nitric oxide promotes seed germination under salt stress. (**a**) Seed germination under different concentrations of salt solution; (**b**) Seed germination rate at 60 h under CK (water) and 100 mM NaCl plus different concentrations of SNP (0, 50, 100, 150 and 200 μM); (**c**) Seed germination rate at 60 h under CK and 100 mM NaCl plus different concentrations of cPTIO (0, 100, 200 and 300 μM). The data are the means of three independent replications (*n* = 50) ± SD. The asterisk (* and **) indicates a significant difference at the *p* < 0.05 and *p* < 0.01 level, respectively, and ns means not significant, by Student’s *t*-test analysis.

Salt stress was demonstrated to significantly inhibit seed germination in many species, such as *Arabidopsis* [43], soybean [12], cotton [44], and *Stylosanthes humilis* [45]. In rice, seed germination was also significantly suppressed by salt stress in a concentration-dependent manner (Figure 1a), in which 100 mM sodium chloride (NaCl) was selected for the further experiments. Similar to other studies, application of SNP, a NO donor, has promoted seed germination of rice under salt stress (Figure 1b). Interestingly, the optimal concentration of SNP to increase seed germination rate under salt stress was 100 μM. In contrast, application of c-PTIO, a NO scavenger, has aggravated the inhibition effect on seed germination in a concentration-dependent manner, too (Figure 1c).

**Figure 2:**
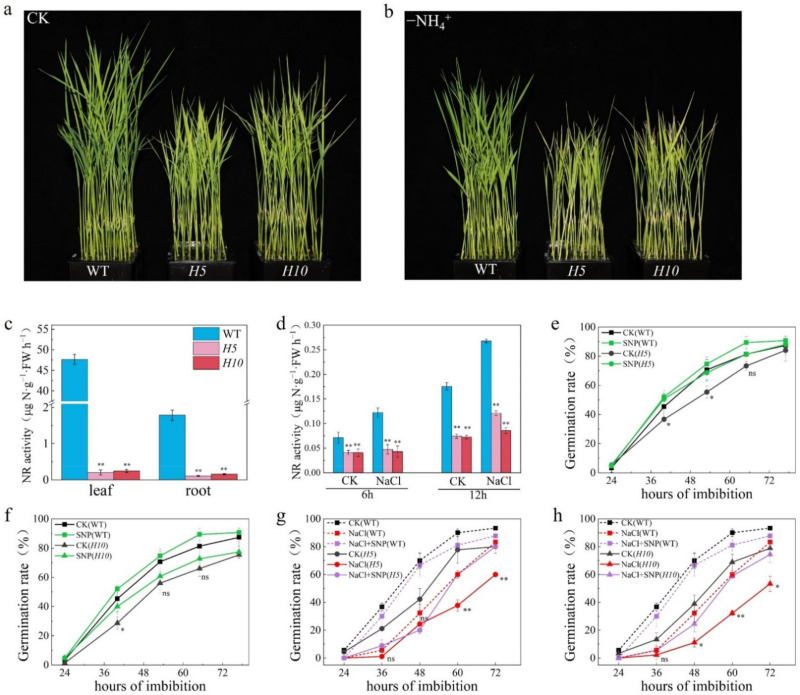
NR-associated NO is involved in enhancing seed germination under salt stress. (**a**) Phenotype of triple mutants, H5 and H10, at 7d-old hydroponic cultured seedlings using Kimura B nutrient solution; (**b**) Phenotype of triple mutants, H5 and H10, at 7d-old hydroponic cultured seedlings using ammonia-deprived nutrient solution; (**c**) NR activity in leaf and root of 14d−old seedlings; (**d**) NR activity in seed germinated under salt stress at 6 h and 12 h. (**e**,**f**) Seed germination of H5 and H10 under CK and 100 μM SNP; (**g**,**h**) Seed germination of H5 and H10 under 100 mM NaCl and 100 mM NaCl plus 1 mM NaNO_3_. The data are the means of three independent replications (*n* = 50) ± SD. The asterisk (* and **) indicates a significant difference at the *p* < 0.05 and *p* < 0.01 level, respectively, and ns means not significant, by Student’s *t*-test analysis.

To further reveal the function of NO in improving salt tolerance during seed germination, a triple mutant of three nitrate reductase encoding genes, *OsNR1*, *OsNR2* and *OsNR3*, was generated by CRISPR/Cas9 (Figure S1). Seedlings of two independent triple mutant lines, H5 and H10, were less vigorous than WT after 10 days of hydroponic culture (Figure 2a). However, the phenotype of triple mutants was similar to WT plant at tillering and grain-filling stage (Figure S2). As expected, H5 and H10 displayed a significantly short and yellowing phenotype when compared with WT plant under ammonium-deprived conditions (Figure 2b), suggesting that utilization of nitrate was blocked in triple mutants. Enzyme activity assays in seedlings under CK condition shown that nitrate reductase (NR) activity was barely detectable both in leaf and root of triple mutants (Figure 2c). In addition, NR activity during seed germination was also significantly lower compared to WT (Figure 2d). Interestingly, NR activity was significantly increased by salt stress during seed germination in WT plant but not in triple mutants which displayed a similar NR activity in salt-treated seed with that in CK condition (Figure 2d), implying that the enzyme NR might play a key role in response to salt stress during seed germination. To answer this, we then carried out seed germination experiments. Our results shown that seed germination was significantly delayed in both triple mutant lines, which was effectively rescued by application of SNP (Figure 2e,f). Furthermore, both triple mutants were much more sensitive to salt stress than WT plant during seed germination. Similarly, application of SNP also promoted seed germination of H5 and H10 (Figure 2g,h), suggesting the NR-associated NO is crucial to promoted seed germination of rice under salt stress.

### 2.2. NR-Associated NO Increased Salt Tolerance by Promoting Expression of ABA Catabolism Genes during Seed Germination under Salt Stress

**Figure 3:**
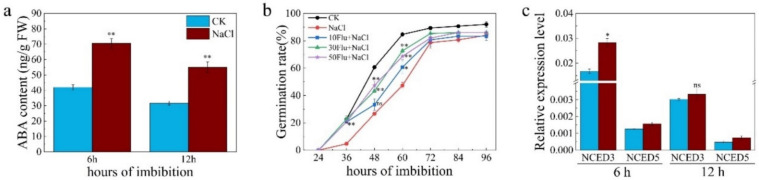
Salt stress increased ABA biosynthesis in both WT and triple mutants during seed germination. (**a**) ABA content of WT seed germinated under water and 100 mM NaCl; (**b**) Seed germination under water and 100 mM NaCl plus different concentrations of fluridone (0, 10, 30 and 50 μM); (**c**) Expressions of *OsNCED3* and *OsNCED5* in WT germinated under water and 100 mM NaCl solution. The data are the means of three independent replications (*n* = 20) ± SD. The asterisk (* and **) indicates a significant difference at the *p* < 0.05 and *p* < 0.01 level, and ns means not significant, respectively, by Student’s *t*-test analysis.

In our previous study, we found that NO was involved in seed dormancy breaking by inducing the expression of *CYP707A2* gene, a key gene for ABA catabolism in *Arabidopsis* [30]. Furthermore, it was reported that ABA biosynthesis was induced by salt stress which thus inhibited seed germination of soybean and *Arabidopsis* [12,43]. Here in rice, we also found that ABA content in seeds under salt stress was higher than that under CK condition at both 6 h and 12 h after imbibition (Figure 3a). Application of fluridone, an ABA biosynthesis inhibitor, can partially rescued seed germination under salt stress (Figure 3b). Then abundances of two key ABA biosynthesis gene, *OsNCED3* and *OsNCED5*, were examined, results shown that *OsNCED3* was significantly induced by salt stress in WT plant at 6 h after imbibition, while *OsNCED5* displayed a similar expression trend with *OsNCED3* but was only slightly induced by salt (Figure 3c). These results suggested that ABA biosynthesis were also induced by salt stress which is in agreement with previous studies.

**Figure 4:**
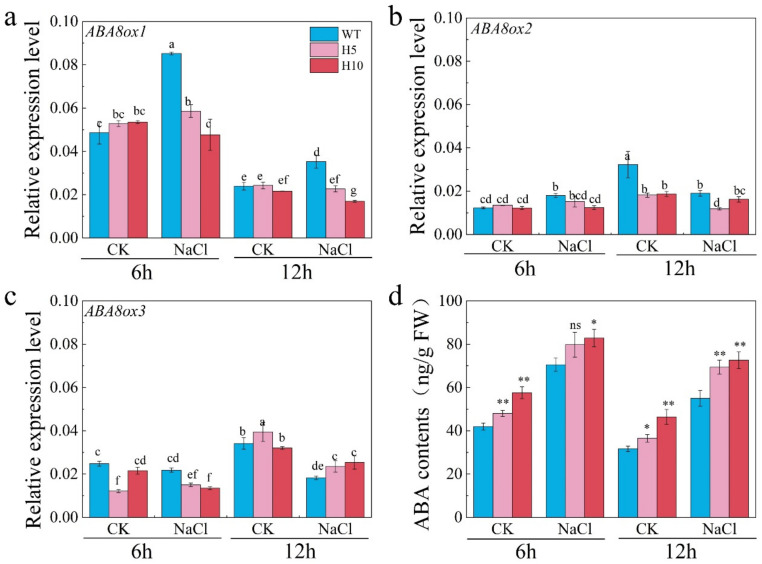
NR-associated NO increased expression of ABA catabolism genes during seed germination under salt stress. (**a**–**c**) Expressions of *OsABA8ox1*, *OsABA8ox2* and *OsABA8ox3* in WT, H5 and H10 germinated under water and 100 mM NaCl solution; (**d**) ABA content of WT and triple mutant seeds germinated under water. The data are the means of three independent replications (*n* = 20) ± SD. The asterisk (* and **) indicates a significant difference at the *p* < 0.05 and *p* < 0.01 level, respectively, and ns means not significant, by Student’s *t*-test. Different letters a, b, c and others indicate significant differences, *p* < 0.05, by Duncan’s test analysis.

ABA level in plant is controlled by the antagonism between biosynthesis and catabolism [11]. To reveal the function of NR-associated NO on ABA catabolism during seed germination under salt stress, the expression of ABA catabolism genes was examined in WT and triple mutant seeds. Results shown that *OsABA8ox1* was induced by salt stress at 6 h after imbibition (Figure 4a). Interestingly, the expression of *OsABA8ox1* was down-regulated in both triple mutants under salt stress condition when compared with WT plant (Figure 4a), which was similar with the NR enzyme activity in WT and triple mutant seed under CK and salt conditions (Figure 2d), suggesting that *OsABA8ox1* might be regulated by NO during seed germination in response to salt stress. Expressions of *OsABA8ox2* and *OsABA8ox3* were not changed by salt stress in both WT and triple mutants (Figure 4b,c). ABA content was higher in both triple mutants than WT seed under CK condition. Under salt stress, ABA content was significantly increased in WT seed and triple mutants at both 6 h and 12 h after imbibition (Figure 4d). These results suggest that NR associated NO plays a key role in inducing the expression of ABA catabolism genes during seed germination under salt stress.

### 2.3. OsNLP2 Was Induced by Salt Stress to Upregulate Expression of OsNRs and Therefore Enhance the NR Activity

**Figure 5:**
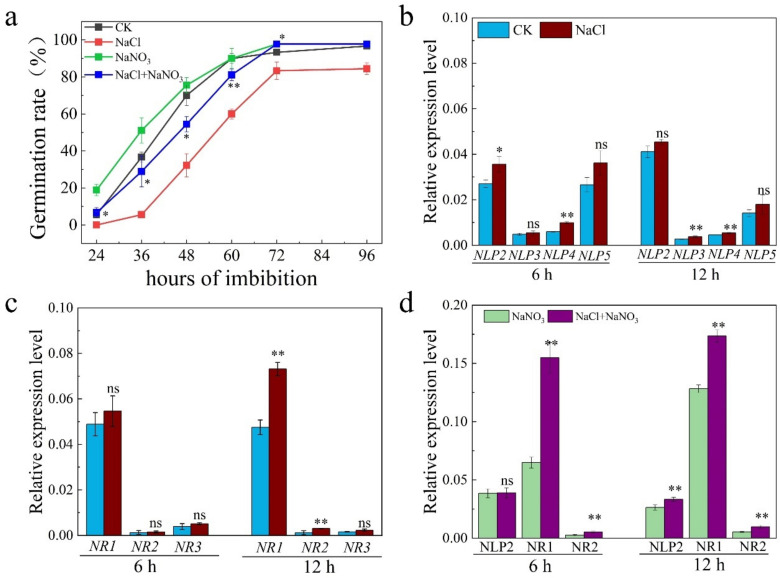
*OsNLP2* was induced by salt stress to promote expression of *OsNR* gene families. (**a**) Seed germination of WT plant under water, 100 mM NaCl, 1 mM NaNO_3_ and NaCl plus NaNO_3_; (**b**,**c**) Expression of *OsNLP* and *OsNR* gene families in WT seed germinated under water and 100 mM NaCl treatments; (**d**) Expression of *OsNLP2*, *OsNR1* and *OsNR2* in WT seed germinated under NaCl and NaCl plus NaNO_3_ treatments. The data are the means of three independent replications (n = 50) ± SD. The asterisk (* and **) indicates a significant difference at the *p* < 0.05 and *p* < 0.01 level, respectively, and ns means not significant, by Student’s *t*-test analysis.

Nitrate was well reported to promote seed dormancy breaking and germination in different species [35]. However, whether and how nitrate is involved in salt response during seed germination is still obscure. In this study, we found that application of 1 mM nitrate could slightly enhance germination rate of *Nipponbare* (WT) seed. Whereas under salt stress, exogenous nitrate has significantly promoted seed germination (Figure 5a). Then we examined the expression of *OsNLP* gene family by qPCR and found that *OsNLP2*, the rice orthologue of *AtNLP8* in *Arabidopsis*, was significantly induced by salt stress at 6 h after imbibition (Figure 5b). Similar to *OsABA8ox1* which is a possible target for NLP2, *OsNR1* and *OsNR2* were also induced by salt stress at 12 h (Figure 5c). Application of nitrate during seed germination under salt stress has significantly increased the expression of *OsNR1* and *OsNR2*, though the expression of *OsNLP2* was not further promoted by nitrate (Figure 5d). Since NLP transcript factors have been well reported to activate the expression of *OsNRs* and *OsNIRs* genes, we thus speculated that NLP2-NR module might be crucial in mediating nitrate-promoted seed germination under salt stress.

**Figure 6:**
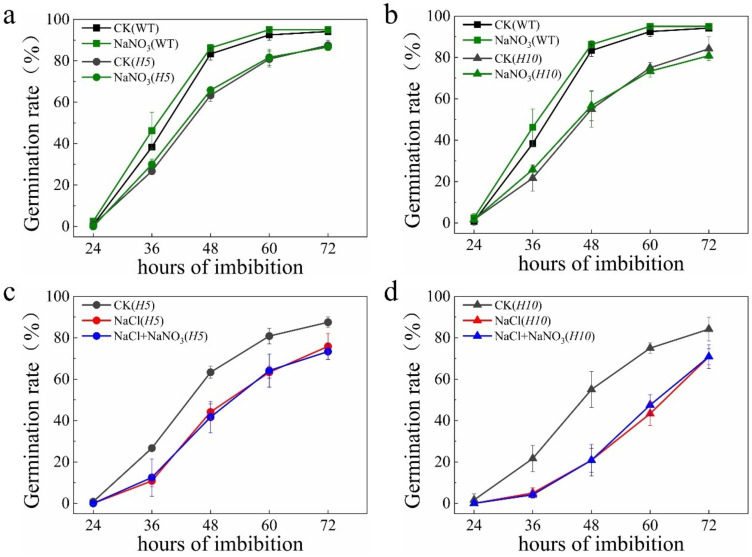
Nitrate-promoted seed germination under salt stress was blocked in triple mutants. (**a**,**b**) Seed germination of H5 and H10 under treatments of water and 1 mM NaNO_3_; (**c**,**d**) Seed germination of H5 and H10 under treatments of NaCl and NaCl plus NaNO_3_. The data are the means of three independent replications (*n* = 50) ± SD.

To address this speculation, we compare the germination rate of WT and triple mutants treated with nitrate. Our results shown that nitrate has slightly promoted seed germination by less than 10% of WT plant (Figure 5a). However, germination of both triple mutants, H5 and H10, were not changed by nitrate (Figure 6a,b), differing from that by SNP (Figure 2e,f). Moreover, application of nitrate to WT seed under salt stress has significantly elevated the seed germination by more than 20% at 72 h after imbibition (Figure 5a). Whereas in the triple mutants, nitrate was still unable to accelerate the germination rate under salt stress condition (Figure 6c,d). These findings further confirm that nitrate promoted salt tolerance during seed germination is mediated by NLP2-NR module.

### 2.4. Nitrate Promoted Seed Germination under Salt Stress Was Partially Blocked in ABA Catabolism Mutants

**Figure 7:**
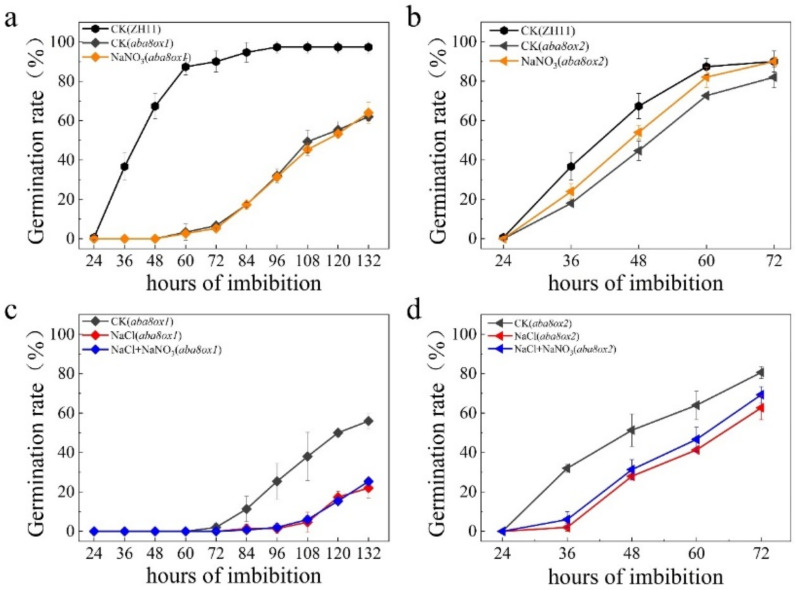
Effect of nitrate on ABA catabolism defective mutants during seed germination under salt stress. (**a**,**b**), Effect of 1 mM NaNO_3_ on seed germination of three mutants of ABA catabolism gene, *aba8ox1* and *aba8ox2*; (**c**,**d**), Seed germination of *aba8ox1* and *aba8ox2* under treatments of NaCl and NaCl plus NaNO_3_. The data are the means of three independent replications (*n* = 50) ± SD.

Both nitrate and NO were proved to induce expression of ABA catabolism gene and thus leads to degradation of ABA. We have generated mutants of ABA catabolism genes, *OsABA8ox1* and *OsABA8ox2*, by CRISPR/Cas9. Germination rates of *aba8ox1* and *aba8ox2*, were delayed compared with WT plant Zhonghua11 (ZH11) (Figure 7a–c), especially the mutant of *aba8ox1*. Germination rate of both mutants were all significantly enhanced by exogenous gibberellin (Figure S3). In addition, application of nitrate could not accelerate seed germination of *aba8ox1* (Figure 7a), but slightly increase germination rate of *aba8ox2* (Figure 7b). In addition, both *aba8ox1* and *aba8ox2* were much more sensitive to salt treatment than WT plant (Figure 1a). Moreover, nitrate application has partially promoted seed germination of *aba8ox2* under salt stress and was still failed to promote seed germination of *aba8ox1* under salt stress (Figure 7c,d). To further confirm this result, the ABA catabolism defective mutants were treated with 100uM SNP, it was shown that seed germination of *aba8ox1* mutant was not promoted by application of SNP (Figure S4a), whereas *aba8ox2* was partially accelerated by SNP during seed germination (Figure S4b). Under salt stress, the promoting effect of SNP on seed germination was further enhanced on *aba8ox2* (Figure S4d). However, germination of *aba8ox1* under salt stress was still unchanged by SNP (Figure S4c), similar with that in CK condition. These results suggest that nitrate promoted seed germination under salt stress was partially blocked in *aba8ox1* mutant.

**Figure 8:**
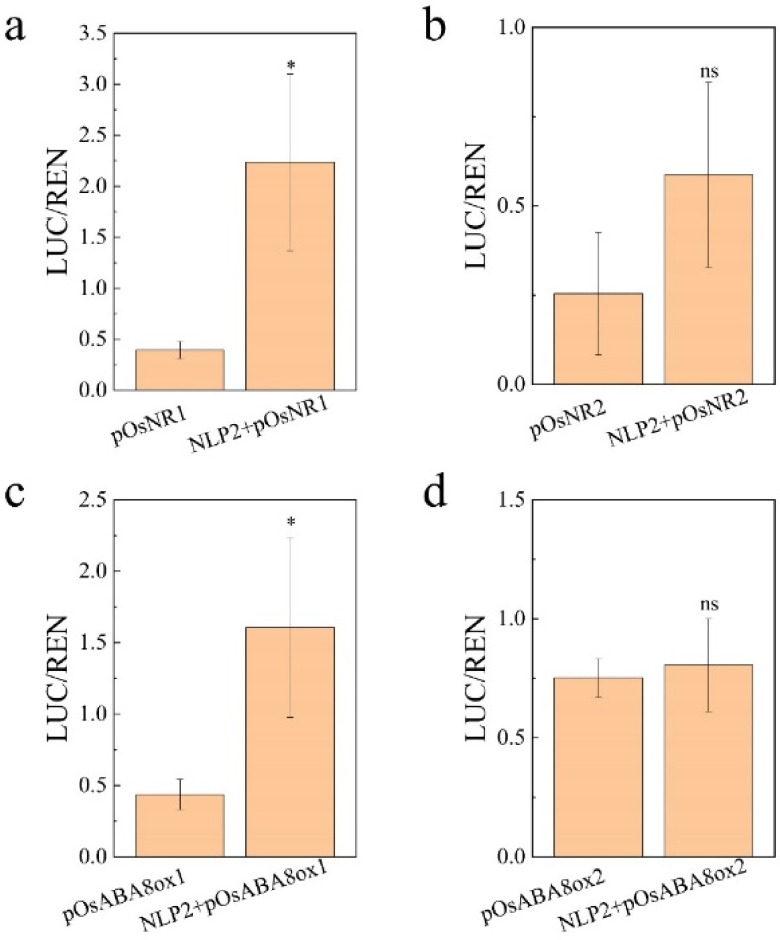
Transient trans-activation expression assays for NLP2 in rice protoplasts. (**a**,**b**) Transient trans-activation for NLP2 on *OsNR1* and *OsNR2* genes; (**c**,**d**) Transient trans-activation for NLP2 on *OsABA8ox1* and *OsABA8ox2* genes. The relative LUC/REN ratio (see Materials and Methods) shown on the x-axes represents the relative promoter activity. The data are the means of three independent replications and error bar indicate ± SD. The asterisk (* and **) indicates a significant difference at the *p* < 0.05 and *p* < 0.01 level, respectively, and ns means not significant, by Student’s *t*-test analysis.

It was showed that OsNLP1, OsNLP3 and OsNLP4 can activate the expression of *OsNR1* and *OsNR2* genes while AtNLP8, a homologous gene of *OsNLP2*, activate the expression of ABA catabolism gene CYP707A2 in Arabidopsis [35,37,38,46]. To further examine the relationship between OsNLP2 and those salt-induced genes, *OsABA8ox1*, *OsNR1* and *OsNR2* (Figure 4b and Figure 5c), we performed transient trans-activation expression assay for OsNLP2 on both ABA catabolism genes and nitrate reductase encoding genes. We found that expression of *OsABA8ox1* and *OsNR1* was significantly activated by OsNLP2 in rice protoplast (Figure 8a,c), and *OsNR2* was also slightly activated by OsNLP2 (Figure 8b). In consistent with the result of nitrate application on *aba8ox2* mutant, OsNLP2 did not activate the expression of *OsABA8ox2* gene rice protoplast (Figure 8d). These results further confirmed that nitrate-activated OsNLP2 regulated the expression of *OsNR1* and *OsABA8ox1* gene to promote seed gemination under salt stress.

## 3. Discussion

Salt stress is well known as one of the most devastating abiotic stress factors in agriculture all over the world. Numerous studies in the past have demonstrated that NO was involved in salt tolerance, acting as ROS scavenger, signaling molecule, and interacting with other molecules [20,47]. Application of SNP has significantly mitigated the effect of salt stress on seedling growth inhibition by increasing the expression of antioxidant-related genes in different rice, especially the salt sensitive cultivar, Ediget [48]. Furthermore, the ROS-dependent lipid peroxidation and protein oxidation by ROS induced by salt stress is effectively inhibited by NO [49]. In soybean, H_2_O_2_ was increased dramatically under long-term salinity stress, which was reduced to the basal level by exogenous NO [50]. Germination, the most important stage of the plant life cycle, is severely repressed by abiotic stresses [51]. Salt stress greatly inhibits seed germination, resulting in a reduction in germination rate and a delay in seedling establishment [52,53], which is also alleviated by application of SNP to the germinating seed under salt stress [24]. It is worth mentioning that the most optimal concentration of SNP with the highest mitigation effect is 50 μM for seed of pakchoi (*Brassica chinensis* L.) [24], which is similar to our result in which 100 μM SNP has the highest alleviation effect of seed germination under salt stress (Figure 1b), suggesting that NO mitigate harmful effect of stress on seed germination in a complicated pattern.

### 3.1. NR-Associated NO Plays a Key Role in Regulating Seed Germination and Salt Tolerance

In plants, there are several NO origins which can be divided into enzymatic pathway and non-enzymatic pathway [20]. Many studies have demonstrated that NR-dependent pathway was the major source for NO production and plays a key role in regulating plant NO homeostasis [54,55]. Both pharmacological and genetical evidence have supported that NR is crucial for NO production in plants [49]. Application of tungstate, sodium azide and potassium cyanide will significantly reduce the NO production in plant [56,57]. While mutation of nitrate reductase genes and *AtNOS1* simultaneously has led to a reduced NO production, inhibition of seed germination and reduced plant size in *Arabidopsis*. Application of SNP was able to significantly, but not fully, promote seed germination of this triple mutant [19]. Here in this study, we found that triple mutant of three nitrate reductase genes in rice displayed a delayed germination phenotype which was also partially rescued by exogenous NO (Figure 2e,f). Our results further supported that NR-associated NO production is crucial in regulating seed germination.

In contrast with NO, the function of NR in salt tolerance has not yet been well demonstrated. Nevertheless, it was reported that NR-dependent NO production was important in response to salt stress in cucumber root when subjected to 50 mM sodium chloride [42] and in soybean during seed germination under 50 mM sodium chloride [41]. However, these results were all based on pharmacological experiment by using sodium tungstate which might have other side effect on plant. Interestingly, deficiency of NO production in the triple mutant of *Arabidopsis* displayed an increased dehydration tolerance by efficiently regulating stomata close when compared with WT plant [19], suggesting that NR might play a negative role in response to water stress. In the present study, we found that NR activity was increased by salt stress in the early stage of seed germination (Figure 2d). In addition, the enhanced NR enzyme activity by salinity was blocked in the triple mutants, H5 and H10 (Figure 2d), resulting in higher sensitivity to salt stress during seed germination than seed of WT plant (Figure 2g,h). Application of SNP to these two triple mutants has significantly reduced the sensitivity and thus promoted seed germination of H5 and H10 under salt stress (Figure 2g,h). This genetic evidence strongly indicates that NR plays a key role in promoting salt tolerance during seed germination of rice by producing NO.

Phytohormone ABA plays a crucial role in salt response by acting as a central integrator that links the complicated developmental processes and salt stress [9]. ABA is significantly induced by salt stress to deal with osmotic stress and oxidative stress [10]. However, the content of ABA should be carefully controlled during seed germination under salt stress because accumulation of ABA will suppress seed germination and even aggravate oxidative stress via ABI4-RbohD pathway [43]. Indeed, ABA content in salt treated seed at 6 h after imbibition was higher than that in CK condition due to the up-regulation of *OsNCED3* gene by salt (Figure 3a,c). In addition, application of ABA biosynthesis inhibitor has mitigated the suppression effect of salinity on seed germination (Figure 3b), indicating that ABA biosynthesis was induced by salt stress. Interestingly, the expression of *OsABA8ox1* gene was also up regulated during seed germination under salt stress in WT plant but not triple mutant (Figure 4a), leading to a higher level of ABA in seed of H10 (Figure 4d). NO production during the early stage of seed germination has induced the expression of *CYP707A2*, a key ABA catabolism gene in *Arabidopsis*, which thereby promoted seed germination [30]. Furthermore, ABA content in plants is controlled by an antagonism between biosynthesis and catabolism under normal condition and abiotic stresses [11]. Since the NR activity was induced by salt stress in seed of WT plant but not triple mutants (Figure 2d), which is highly similar to the expression pattern of *OsABA8ox1* (Figure 4a), we speculate that NR-dependent NO production was induced by salt stress to up-regulate the expression of *OsABA8ox1* and thereby finely control ABA content in WT seed.

### 3.2. OsNLP2 Was Involved in Salt Defense by Up-Regulating Expression of Both OsNR1 and OsABA8ox1 Genes

NIN-like protein (NLP) belongs to the transcript factor family which act as master regulator in nitrate response by binding to nitrate responsive cis-element (NRE) in many nitrogen responding genes in *Arabidopsis* [58,59]. In rice, 6 *NLP* genes has been identified based on in silico analysis [21]. *OsNLP1*, *OsNLP3* and *OsNLP4* were reported to play key role in improving yield and nitrogen use efficiency. Over expression of these genes all displayed higher yield per plant by increasing tiller number and grain number per plant. What is more, three *OsNLPs* were all found to bind the promoter of *OsNR1*, *OsNR2* and *OsNiR* which thereby regulate the expression of these genes [37,38,46]. Their results strongly indicated that *OsNLPs* might be involved in regulating NO production via NR-NiR pathway. Although functions of *OsNLPs* have been well illustrated in nitrogen response, whether their play a role in stress defense has not yet been reported. In this study, we found salt stress has induced the expression of *OsNLP2* gene at early stage of seed germination (Figure 5b), accompanied by up-regulation of *OsNR1/2* (Figure 5c) and increased NR activity (Figure 2d). Similar with *OsNLP1*, *OsNLP3* and *OsNLP4* genes, *OsNLP2* was also found to regulate the expression of *OsNR1* and *OsNR2* in our transient trans-activation expression assay (Figure 8a,b). Together, these results further proved that *OsNLP2* is involved in NO production via regulating expression of *OsNRs* in response to salt stress.

Nitrate has been well known to promote seed dormancy breaking and germination [60]. *AtNLP8*, as a nitrate responsive gene, was activated by application of nitrate and thus increased the expression of *CYP707A2* gene during seed germination in Arabidopsis [35]. In addition, *CYP707A2* plays a key role in regulating seed germination and is activated by NO during seed germination in *Arabidopsis* [30,61], implying that nitrate-activated NLP8 might reduce ABA level by directly binding to promoter of *CYP707A2* and indirectly inducing the expression of *CYP707A2* via enhancing NO production during seed germination in Arabidopsis because the expression of *AtNIA2*, orthologue of *OsNR2*, was induced by nitrate and blocked in *nlp8* mutant. In the present study, it is demonstrated that application of nitrate has significantly alleviated the inhibition effect of salinity on seed germination (Figure 5a). Expression of *OsNR1* and *OsNR2* was further induced by nitrate during seed germination under salt stress (Figure 5d). However, the promoting effect of nitrate on seed germination was blocked in triple mutants under both CK and salt stress conditions (Figure 6), suggesting nitrate-promoted seed germination was partially mediated by NLP2-NR module-dependent NO production. In addition, expression of *OsABA8ox1* was induced by salt stress in WT plant, which is partially reduced in both H5 and H10 under CK or salinity conditions (Figure 4a). Application of nitrate to mutants of ABA catabolism genes and its corresponding wide type plant, Zhong Hua 11 (ZH11), shown that seed of *aba8ox1* was insensitive to nitrate (Figure 7a,c), while *aba8ox2* was partially promoted by nitrate during seed germination (Figure 7b–d). Luciferase assay further revealed that OsNLP2 activated the expression of *OsABA8ox1* gene in response to nitrate application during seed germination under salt stress (Figure 8c).

## 4. Materials and Methods

### 4.1. Plant Materials and Germination Condition

Two japonica rice, Nipponbare and Zhonghua 11, were used in this study. Triple mutants of three nitrate reductase encoding genes, H5 and H10, were generated in the background of Nipponbare, which was referred to WT in this study. Mutants of ABA catabolism genes, *aba8ox1*, *aba8ox2* and *aba8ox3*, were generated in the background of Zhonghua 11 which was referred to ZH11 when using these ABA catabolism mutants in this study. All mutants were generated by CRISPR/Cas9 technology. The mutation patterns of all mutants were shown in Figure S1.

Dehulled rice seeds, the caryopses, were surface-sterilized with 10% sodium hypochlorite for 10 min and washed at least four times with sterile water. The sterile seeds were sown directly on two layers of filter paper in germinating box (10 × 10 cm) with different concentrations of NaCl, Sodium Nitroprusside (SNP) and 1 mM Sodium Nitrate accordingly (presented in the Figure Legend). Seeds were placed in a growth chamber in continuous darkness at 28 °C to facilitate germination. Germination (based on radicles > 3 mm) was recorded every 12 h or daily, depending on the experiment. For each germination test, approximately 50 seeds per genotype were used, and three experimental replications were performed. The average ± SE (standard error) of triplicate experiments were calculated.

### 4.2. RNA Isolation and Quantitative Real-Time PCR

Germinating seeds at different time points were sampled and stored in liquid nitrogen immediately for gene expression and enzyme activity analysis. Total RNA was extracted using Plant RNA Purification Reagent for plant tissue according to the manufacturer’s instructions (Invitrogen) and genomic DNA was removed using DNaseI (TaKara). Then RNA quality was determined by 2100 Bioanalyser (Agilent) and quantified using the ND-2000 (NanoDrop Technologies). Quantitative real-time PCR was performed using the 2× SYBR Green qPCR Master Mix (Yeasen Biotechnology) using a StepOnePlus™ Real-Time PCR System (Applied Biosystems). Gene expression was quantified at the logarithmic phase using the expression of the housekeeping gene *ACTIN7* as an internal control. Three biological replicates were performed for each experiment. Primer sequences for qRT-PCR are shown in Supplementary Table S1.

### 4.3. NR Enzyme Activity Assay

NR was extracted and its activity was assayed according to Scholl et al. [62], which has been widely used to determine the total activity of NR. The extraction buffer consisted of 50 mM phosphate buffer (pH 7.5), 1 mM EDTA and 3 mM cysteine. Seed samples (0.2–0.3 g) were homogenized in a mortar with cold, 1 mL extraction buffer, on ice. The extract was centrifuged for 10 min at 4 °C. 50 μL of enzyme extract was added to 450 μL of reaction buffer (350 μL of 50 mM phosphate buffer, pH 7.5, 50 μL of 0.2 M potassium nitrate, 50 μL of 5 mM NADH), which was beforehand pre-incubated at 30 °C. Blank assays were the same except that NADH was replaced by water. After 15 min, the reaction was terminated by adding 50 μL of 0.6 M zinc acetate. The mixture was then centrifuged, and the supernatant was transferred to a new tube and 50 μL of 12 mM phenazine methosulfate was added. Ten minutes later, 500 μL of 0.02% N-(lnaphthyl) ethylenediaminedihydrochloride and 500 μL of 1% sulfanilamide (dissolved in 3 M HCl) were added. After incubation for 30 min, A540 was measured and the activity was calculated based on a standard curve.

### 4.4. Detection of ABA Content

ABA content was detected by Wuhan MetWare Biotechnology Co., Ltd. (Wuhan, China). Seed samples were frozen in liquid nitrogen, ground into powder, and extracted with methanol/water/formic acid (15:4:1, *v*/*v*/*v*). The combined extracts were evaporated to dryness under nitrogen gas stream, reconstituted in 80% (*v*/*v*) methanol, and filtrated (polytertrafluoroethylene, 0.22 μm; Anpel). The sample extracts were analyzed using an LC-ESI-MS/MS system (HPLC, Shim-pack UFLC SHIMADZU CBM30A system; MS, Applied Biosystems 6500 Triple Quadrupole).

### 4.5. Analysis of Luciferase In Vivo

The 2000-bp sequence of the native *OsNR1*, *OsNR2*, *OsABA8ox1* and *OsABA8ox2* promoters, were amplified from japonica rice genomic DNA. The amplified promoters were cloned into the pGREENII-0080-luc vector by a one-step cloning kit (Vazyme, Nanjing, China) to form the reporter construct. Then, the CDS region of the *OsNLP2* was amplified and cloned into the pGREENII-62-SK vector by the one-step cloning kit (Vazyme) to form the effector construct. Finally, the two constructed vectors were mixed well for the transient expression assay in the rice protoplast. This transient expression assay was performed as described previously [63].

### 4.6. Statistical Analysis

The SPSS 23 software (SPSS Inc., Chicago, IL, USA) was used to analyze significance, data was presented as mean ± SE. Post-hoc comparisons were tested using the Student’s *t*-test, *p* < 0.05 or *p* < 0.01 was considered to be statistically significant.

### 4.7. Accession Numbers

Genes and their accession number used in this study are shown as followed: *OsNR1*, LOC_Os08g36480; *OsNR2*, LOC_Os02g53130; *OsNR3*, LOC_Os08g36500; *OsABA8ox1*, LOC_Os02g47470; *OsABA8ox2*, LOC_Os08g36860; *OsABA8ox3*, LOC_Os09g28390; *OsNLP1*, LOC_Os03g03900; *OsNLP2*, LOC_Os04g41850; *OsNLP3*, LOC_Os01g13540; *OsNLP4*, LOC_Os09g37710; *OsNLP5*, LOC_Os11g16290; *OsNLP6*, LOC_Os02g04340; *Actin7*, LOC_Os01g64630.

## 5. Conclusions

**Figure 9:**
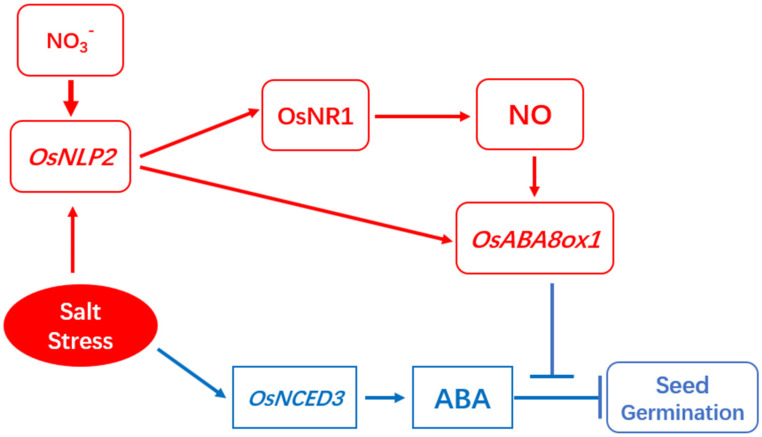
A simplified model of OsNLP2-NR module promotes seed germination under salt stress. Seed germination was significantly reduced by salt stress by promoting ABA biosynthesis and ROS accumulation. To cope with this problem, rice seed has elevated the expression of *OsNLP2* which is also activated by nitrate. The activated OsNLP2 thereby promote the expression of both *OsABA8ox1* and OsNR1/2, resulting in an enhanced ABA catabolism and accumulation of NO production.

In conclusion, our results demonstrate that nitrate responsive gene, *OsNLP2*, plays key role in regulating ABA content by NLP2-NR module-dependent NO production and by activating the expression of *OsABA8ox1* gene during seed germination under salt stress (Figure 9). Seed germination was significantly reduced by salt stress by promoting ABA biosynthesis and ROS accumulation. The expression of OsNLP2 was induced by salt stress during seed germination, which thereby promote the expression of both *OsABA8ox1* and OsNR1/2, resulting in an enhanced ABA catabolism and accumulation of NO production. The elevated NR-dependent NO will then mitigate the inhibition effect of salinity by further promoting ABA catabolism.

## Data Availability

Not applicable.

## Supplementary Materials

The following supporting information can be downloaded at: https://www.mdpi.com/article/10.3390/plants11060795/s1, Figure S1 Mutation patterns of mutants used in this study. a, Sequencing results for mutation position of triple mutants, H5 and H10; b–d, Sequencing results for mutation position of *aba8ox1* and *aba8ox2*; Figure S2 Phenotypes of WT and triple mutant at other developmental stages. a, tilling stage; b, early stage of grain filling; c, mature stage; Figure S3 Seed gemination of ABA catabolism gene mutants under treatment of water and 5 μM GA. a, *aba8ox1*; b, *aba8ox2*. The data are the means of three independent replications (*n* = 50) ± SD; Figure S4 Application of SNP on *aba8ox1* and *aba8ox2* during seed germination. a and b, Seed germination of *aba8ox1* and *aba8ox2* under water and 100 μM SNP treatments; c and d, Seed germination of *aba8ox1* and *aba8ox2* under treatments of NaCl and NaCl plus SNP. The data are the means of three independent replications (*n* = 50) ± SD; Table S1 Primer sequences for qRT-PCR and of luciferase assay in vivo used in this study.
