# Supplementary material for: NLP2-NR Module Associated NO Is Involved in Regulating Seed Germination in Rice under Salt Stress
Source: Plants (Basel). 2022 Mar 17;11(6):795. doi: 10.3390/plants11060795 (PMC8953764; doi:10.3390/plants11060795)
Supplement: Supplementary file 1 [file plants-11-00795-s001.zip › plants-1628416-supplementary.pdf]

NLP2-NR module associated NO is involved in regulating seed germination in rice under salt stress

Yake Yi[a,1], Yaqiong Peng[a,1], Tao Song[b,1], Siqiong Lu[a], Zhenning Teng[a], Qin Zheng[a], Fankai Zhao[a], Shuan Meng[a], Bohang Liu[a], Yan Peng[a], Guanghui Chen[a, *], Jianhua Zhang[c, d, *], Nenghui Ye[a, e, *]

Supplementary data

Table S1 Primer sequences for qRT-PCR and of luciferase assay *in vivo* used in this study.

Figure S1 Mutation patterns of mutants used in this study. a, Sequencing results for mutation position of triple mutants, H5 and H10; b-d, Sequencing results for mutation position of *aba8ox1*and *aba8ox2*.

Figure S2 Phenotypes of WT and triple mutant at other developmental stages. a, tilling stage; b, early stage of grain filling; c, mature stage.

Figure S3 Seed gemination of ABA catabolism gene mutants under treatment of water and 5μM GA. a, *aba8ox1*; b, *aba8ox2*. The data are the means of three independent replications (n=50) ± SD.

Figure S4 Application of SNP on *aba8ox1* and *aba8ox2* during seed germination. a and b, Seed germination of *aba8ox1* and *aba8ox2* under water and 100μM SNP treatments; c and d, Seed germination of *aba8ox1* and *aba8ox2* under treatments of NaCl and NaCl plus SNP. The data are the means of three independent replications (n=50) ± SD.

Table S1 Primer sequences for qRT-PCR and of luciferase assay *in vivo* used in this study.

**Primer nane**	**Sequence (5`-3`)**
qActin7-F	CGGTAATGTGGTCCTTAGTGG
qActin7-R	GGAACCACCAATCCAGACACT
qNR1-F	ATAGGCCATGATTTCCCTTTC
qNR1-R	CTTTACCTCCCATGGCTAAC
qNR3-F	GCCATGATTTCCCTTTCTAAG
qNR3-R	CCGAGTTCTTCCTCTGTTTC
qNR2-F	AGGCCGACCAAGTACGGAAA
qNR2-R	CCTGCACGTCTTCGTCTTCA
qNLP2-R	TCTGTTGTTCTCCACTGCCTTTGC
qNLP2-F	TGCTGCTGTTGCCATTCGTCTAAG
qNLP3-R	TGAGCCACGACGACGACGAG
qNLP3-F	GGAAGGAGGAGGAGGAGGAATCG
qNLP4-R	CGCTCGGCTGAATCCACCATG
qNLP4-F	ACGCTCACCCAACTTCTTTGCTAC
qNLP5-R	AGAACGGCTGGTCAGAGGTAGTC
qNLP5-F	GGTGGCGAAGCGATCCTTGTG
qABA8ox1-F	AAGCTGGCAAAACCAACATC
qABA8ox1-R	CCGTGCTAATACGGAATCCA
qABA8ox2-2-F	ACACTCACGCATCAGGTTATAC
qABA8ox2-2-R	GGGTTGTGATGGATGTTCCT
qABA8ox3-F1	AGTACAGCCCATTCCCTGTG
qABA8ox3-R1	ACGCCTAATCAAACCATTGC
qNCED3-F1	CCCCTCCCAAACCATCCAAACCGA
qNCED3-R1	TGTGAGCATATCCTGGCGTCGTGA
qNCED5-F	ACATCCGAGCTCCTCGTCGTGAA
qNCED5-R	TTGGAAGGTGTTTTGGAATGAACCA
Luc-seq-F	GTGCTGCAAGGCGATTAAG
Luc-seq-R	TTCCAGCGGATAGAATGG
LUC-frame-F1	TCAAATCATTCCGGATACTGCG
REN-frame-F2	ATTGAGCCAGTAGCGCGG
LUC-check-F1	TGAACCATTCAAAGAGAAAGGTG
LUC-check-R1	ACTGCATACGACGATTCTGTGA
SK-seq-F	TCTAGAACTAGTGGATC
SK-seq-R	CGAGGTCGACGGTATCG
pNR1-LUCF	TATAGGGCGAATTGGGTACCCCGTGTATAGCAAAGTCCG
pNR1-LUCR	GCTCTAGAACTAGTGGATCCTTTAGTGCTCGGTTCTAGC
pABA8ox1-LUCF	TATAGGGCGAATTGGGTACCCACAAAGGGTAGCCCCCCAT
pABA8ox1-LUCR	GCTCTAGAACTAGTGGATCCGGCGCCTGCTCACTTCAC
pABA8ox2-LUCF	TATAGGGCGAATTGGGTACCGGAAAGAGGTAGAGAGAGGTGAGGGG
pABA8ox2-LUCR	GCTCTAGAACTAGTGGATCCTGTGTGTCTCCTTGTCCAAACAACTC

pNR2-LUCF	TATAGGGCGAATTGGGTACCTAGAGACCATATATAAATAC
pNR2-LUCR	GCTCTAGAACTAGTGGATCCGTTGCCGATGGAAGGAC
SK-NLP2F	GCTCTAGAACTAGTGGATCCAGTGGATAAGTCGTAAGG
SK-NLP2R	GACGGTATCGATAAGCTTCAGTTGAGGGTTGGGTGA

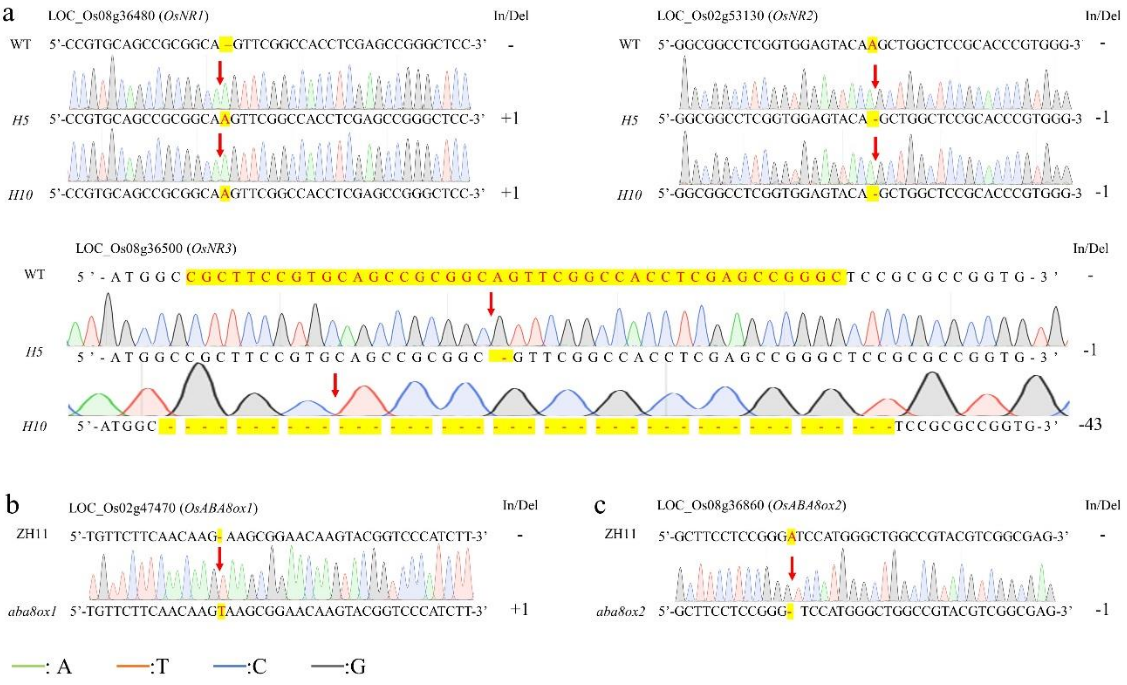

Figure S1 Mutation patterns of mutants used in this study. a, Sequencing results for mutation position of triple mutants, H5 and H10; b-d, Sequencing results for mutation position of *aba8ox1*and *aba8ox2*.

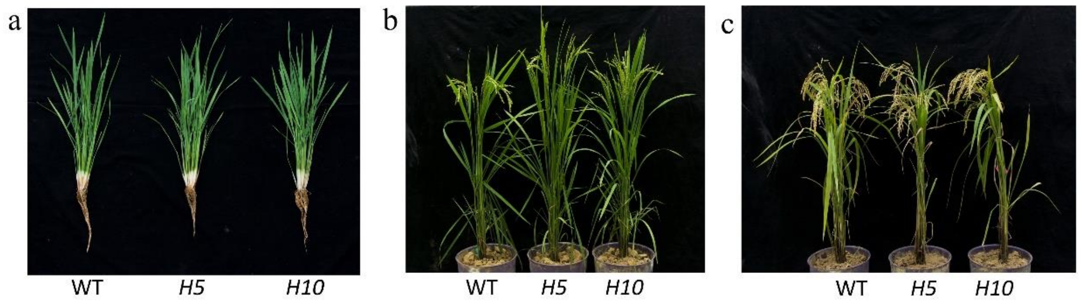

Figure S2 Phenotypes of WT and triple mutant at other developmental stages. a, tilling stage; b, early stage of grain filling; c, mature stage.

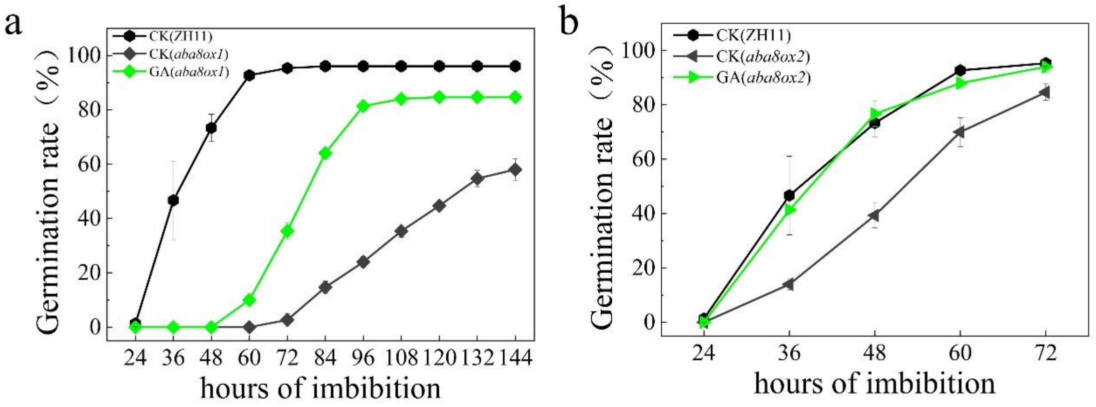

Figure S3 Seed gemination of ABA catabolism gene mutants under treatment of water and 5μM GA. a, *aba8ox1*; b, *aba8ox2*. The data are the means of three independent replications (n=50) ± SD.

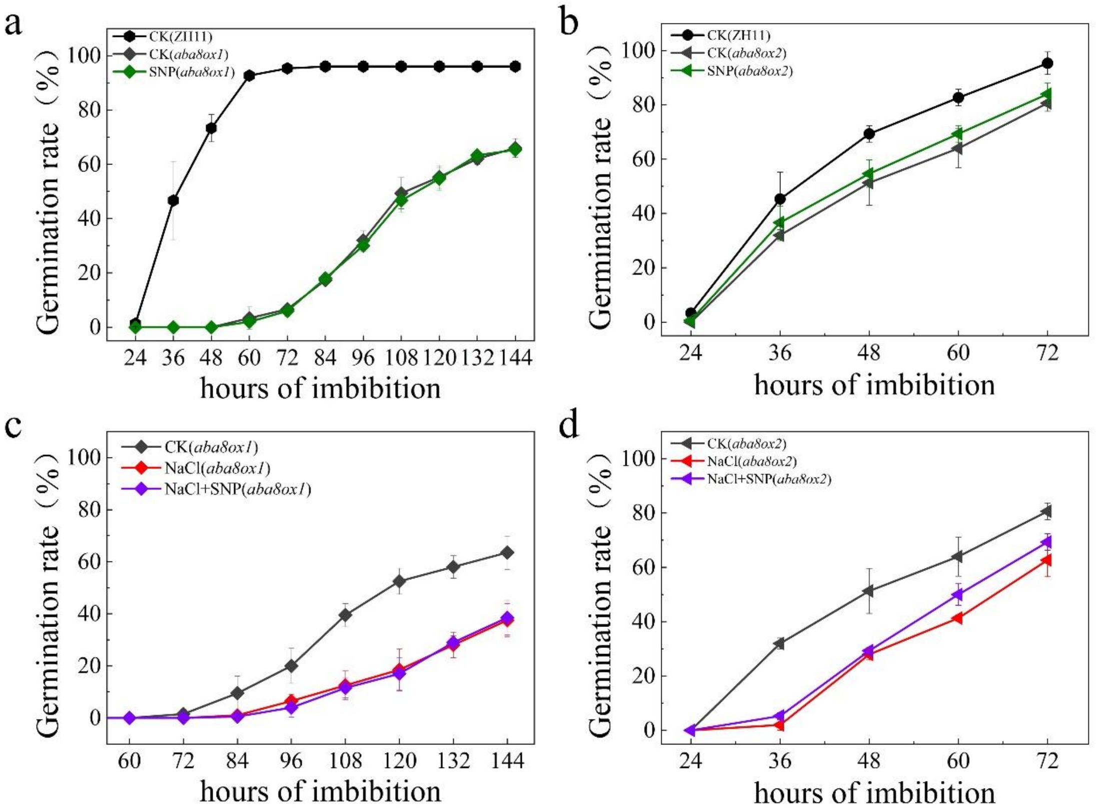

Figure S4 Application of SNP on *aba8ox1* and *aba8ox2* during seed germination. a and b, Seed germination of *aba8ox1* and *aba8ox2* under water and 100μM SNP treatments; c and d, Seed germination of *aba8ox1* and *aba8ox2* under treatments of NaCl and NaCl plus SNP. The data are the means of three independent replications (n=50) ± SD.
